# Chalcone-Derived Lactones: Synthesis, Whole-Cell Biotransformation, and Evaluation of Their Antibacterial and Antifungal Activity

**DOI:** 10.3390/molecules28093800

**Published:** 2023-04-28

**Authors:** Witold Gładkowski, Monika Siepka, Barbara Żarowska, Agata Białońska, Barbara Gawdzik, Mariusz Urbaniak, Czesław Wawrzeńczyk

**Affiliations:** 1 Department of Food Chemistry and Biocatalysis, Wrocław University of Environmental and Life Sciences, Norwida 25, 50-375 Wrocław, Poland; monikasiepka@yahoo.com; 2 Department of Biotechnology and Food Microbiology, Wrocław University of Environmental and Life Sciences, Chełmońskiego 37/41, 51-630 Wrocław, Poland; barbara.zarowska@upwr.edu.pl; 3 Department of Crystallography, University of Wrocław, Joliot Curie 14, 50-383 Wrocław, Poland; agata.bialonska@chem.uni.wroc.pl; 4 Institute of Chemistry, Jan Kochanowski University, Świętokrzyska 15 G, 25-406 Kielce, Poland; b.gawdzik@ujk.edu.pl (B.G.); mariusz.urbaniak@ujk.edu.pl (M.U.)

**Keywords:** chalcone, bromolactonization, hydroxylactones, microbial dehalogenation, translactonization, antibacterial activity, antifungal activity

## Abstract

Four compounds with lactone moiety were synthesized from chalcone **1** in three- or four-step synthesis. γ-Bromo-δ-lactone **5** was the only product of bromolactonization of acid **4** whereas bromolactonization of ester **3**, apart from lactone **5** also afforded its isomer **6** and two diastereoisomeric δ-hydroxy-γ-lactones **7** and **8**. Lactone **8** was also obtained in 88% yield as a product of simultaneous dehalogenation and translactonization of γ-bromo-δ-lactone **5** by *Penicillum frequentans* AM 359. Chalcone-derived lactones **5**–**8** were subjected to the tests on antimicrobial activity and the results compared with activity of starting chalcone **1**. Obtained lactones **5**–**8** in most cases limited the growth of tested bacterial and fungal strains. The highest activity was found for δ-hydroxy-γ-lactone **8** which completely inhibited the growth of *Staphylococcus aureus, Fusarium graminearum*, *Aspergillus niger*, and *Alternaria* sp. The introduction of lactone moiety into chalcone scaffold significantly improved antimicrobial activity of the compound: γ-bromo-δ-lactone **6** and δ-hydroxy-γ-lactone **8** were significantly stronger growth inhibitors of *S. aureus* and *F. graminearum*. In the case of the latter, a clear positive effect of the lactone function on the antifungal activity was also observed for γ-bromo-δ-lactone **5**.

## 1. Introduction

Chalcones (1,3-diarylprop-2-en-1-ones) are secondary metabolites of plants which belong to the class of flavonoids. They are widely distributed in vegetables, fruits, and teas [1]. Some plants rich in chalcones from the *Glycyrrhiza*, *Piper,* or *Angelica* genus have been used for many years as therapeutic agents in Balkan countries [2]. Indeed, for natural and synthetic chalcones, a number of studies have shown a wide spectrum of biological activity of such as antioxidant, anticancer, anti-inflammatory, antidiabetic, antiviral, or antiparasitic [2,3,4,5]. Several chalcone derivatives are known to be the commercial drugs, *i.a.,* choleretic drug metochalcone or antiulcer and mucoprotective drug sofalcone [6].

One of the most studied bioactivities of natural and synthetic chalcones are their antibacterial [7] and antifungal [8] properties. Numerous chalcones exhibiting these activities can be found in literature, including those synthesized by Claisen–Schmidt condensation of substituted benzaldehydes with acetophenone [9,10,11], with cyclopentanone or cyclohexanone [12] or with 1-tetralone [13]. Another example of chalcones with antimicrobial activity are those based on thiazole [14] or chromone scaffold [15]. The simplest chalcone, 1,3-diphenylprop-2-en-1-one (**1**), exhibited strong antifungal effects against some dermatophytes [16].

Our research group was also involved in the studies on the lactones with antimicrobial activity, derived from simple aromatic aldehydes [17,18,19] or β-cyclocitral [20]. In one of our last papers, we presented the results of the studies on the antimicrobial properties of ε-lactones obtained from flavanones by Baeyer–Villiger oxidation. It has been confirmed that introduction of lactone moiety increased the antimicrobial activity of starting flavanones [21]. In this work, we would like to investigate if the lactones obtained from chalcone **1** by synthetic and biotechnological methods exhibit higher antimicrobial properties than starting substrate.

## 2. Results and Discussion

### 2.1. Synthesis

**Scheme 1:**
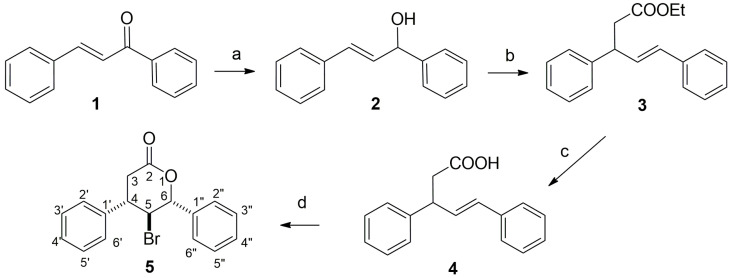
Synthesis of γ-bromo-δ-lactone **5** from chalcone **1** involving halolactonization of acid **4**. Reactions and conditions: (a) NaBH_4_, MeOH/H_2_O, 0 °C, (b) CH_3_C(OEt)_3_, EtCOOH, 138 °C, (c) NaOH, EtOH, reflux (d) NBS, THF, AcOH, rt.

Direct substrates for halolactonizations were acid **4** or ethyl ester **3**. Both substrates were obtained from chalcone **1** in two or three-step procedure presented on Scheme 1. In the first step, chalcone **1** was reduced with sodium borohydride in methanol–water solution (10:1) at 0 °C to afford racemic alcohol **2** in 97% yield. Alcohol **2** was then subjected to the Claisen rearrangement with triethyl orthoacetate in the presence of catalytic amount of propionic acid to obtain ethyl ester **3** in 70% yield. Ester **3** was subsequently hydrolyzed with NaOH in EtOH to afford acid **4** in 81% yield. All spectroscopic data of alcohol **2**, ester **3,** and acid **4** were in accordance with those reported in literature [22,23].

**Figure 1:**
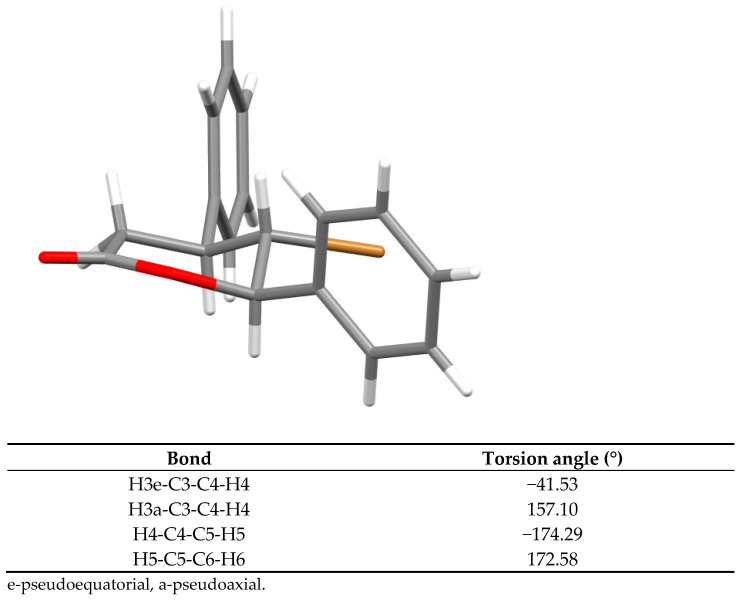
Crystal structure of γ-bromo-δ-lactone **5** and values of selected torsion angles.

**Scheme 2:**
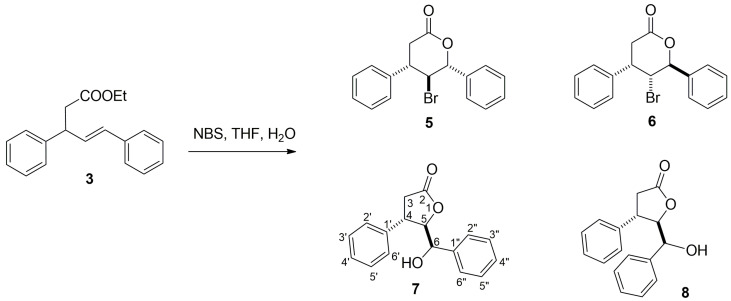
Bromolactonization of ester **3**.

Bromolactonization of acid **4** with N-bromosuccinimide (NBS) in tetrahydrofurane (THF) afforded γ-bromo-δ-lactone **5** as the only product in 37% yield (Scheme 2). Its structure was confirmed by spectroscopic data. On IR spectrum, the absorption bands at 1729 and 1249 cm^−1^ of corresponding C=O and C-O bonds confirmed the presence of δ-lactone ring in the molecule. On ^1^H NMR, two diastereotopic protons of CH_2_-3 group were represented by two doublets of doublets at 2.90 ppm (*J* = 17.8 and 10.1 Hz) and 3.19 ppm (*J* = 17.8 and 6.7 Hz). The values of the smaller coupling constants in these multiplets let us assign the multiplet located at higher field to pseudoaxial proton and the multiplet located at lower field to the pseudoequatorial one. Proton H-4 gave triplet (*J* = 10.1 Hz) of doublets (*J* = 6.7 Hz) at 3.63 ppm, proton H-5 triplet (*J* = 10.1 Hz) at 4.30 ppm, and the signal of proton H-6 was recognized as doublet at 5.47 ppm (*J* = 10.1 Hz). The coupling constant value (10.1 Hz) between proton H-5 and H-4 as well as H-5 and H-6 indicated the pseudoaxial orientations of these protons and thus the pseudoequatorial orientations of bromine and two phenyl rings. These data were fully confirmed by X-ray analysis. Crystal structure of compound **5**, shown in Figure 1, revealed *trans* orientation of bromine at C-5 in relation to phenyl substituents at C-4 and C-6 as well as the half-chair conformation of δ-lactone ring, assumed on the basis of spectroscopic data. The values of torsion angles between corresponding bonds were compatible with the coupling constants found in the ^1^H NMR spectrum. The spectroscopic data of lactone **5** were consistent with those obtained for its enantiomer 4*R*,5*R*,6*S*, an intermediate in the synthesis of (˗)-clausenamide, exhibiting significant neuroprotective effect against β-amyloid in cellular models [23].

Searching for new methods to improve the yield of lactone **5**, bromolactonization of ester **3** using NBS in THF/H_2_O solution was carried out (Scheme 2) according to the procedure described by Obara et al. [24]. As a result, we obtained bromolactone **5** as a major product with a significantly higher isolated yield (79%) compared to the yield observed for the bromolactonization of acid **3**. Simultaneously, three new lactones, not described previously, were formed as minor products: bromolactone **6** (4% isolated yield) and two δ-hydroxy-γ-lactones **7** and **8**, obtained in 3 and 6% isolated yields, respectively.

**Figure 2:**
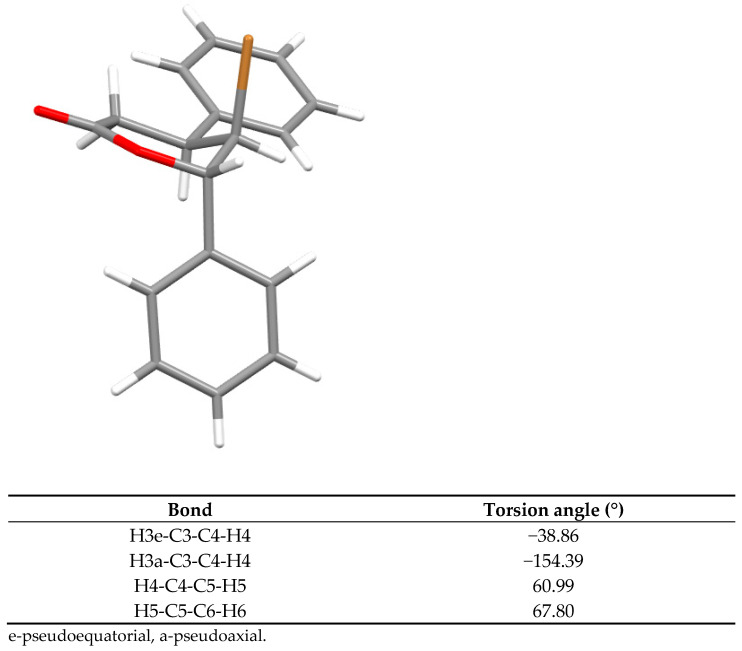
Crystal structure of γ-bromo-δ-lactone **6** and values of selected torsion angles.

Spectral data obtained for lactone **6** together with X-ray analysis allowed us to establish the structure of this product. The presence of δ-lactone ring was confirmed by absorption bands at 1730 and 1205 cm^−1^ on the IR spectrum. The orientations of substituents at the lactone ring were clearly indicated by the crystal structure (Figure 2).

One can see pseudoaxial orientations of bromine atom at C-5 and phenyl ring at C-6 as well as pseudoequatorial orientation of phenyl ring at C-4, which contrary to the lactone **5** was *cis* oriented to the bromine atom. The torsion angles between proton H-5 and protons H-4 and H-6 (Figure 2) were in accordance with the corresponding coupling constants found in ^1^H NMR spectrum. Similar to lactone **5**, the signals of H-5 and H-6 were also triplet (4.71 ppm) and doublet (5.97 ppm), respectively, but with small coupling constant (*J* = 2.5 Hz) resulting from the pseudoequatorial orientations of these protons.

**Figure 3:**
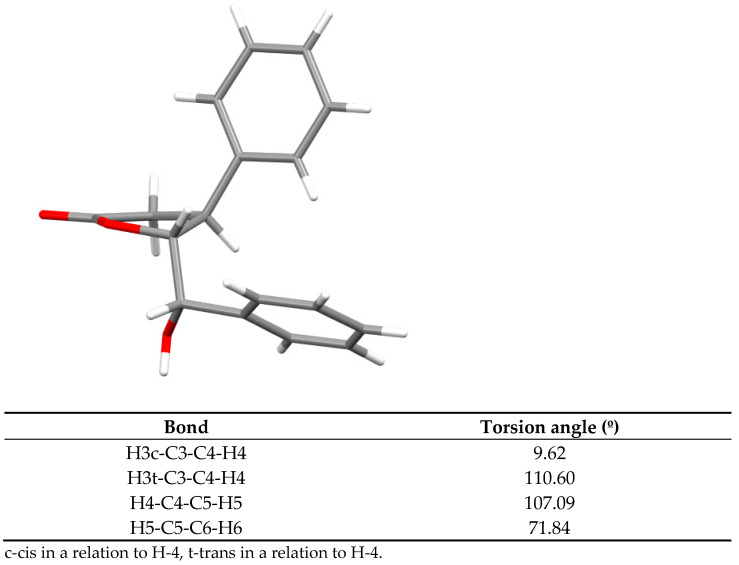
Crystal structure of *trans* δ-hydroxy-γ-lactone **7** and values of selected torsion angles.

Based on the previous investigations on the bromolactonization of γ,δ-unsaturated acids [24,25,26], we also expected the formation of two diastereoisomeric δ-bromo-γ-lactones. Indeed, two other products were isolated from the products mixture but surprisingly, their structural X-ray analysis did not confirm their structures as predicted *cis* and *trans* isomers of δ-bromo-γ-lactone. The crystal structure of compound **7** (Figure 3) undoubtedly showed that the isolated compound was *trans* δ-hydroxy-γ-lactone.

On the IR spectrum of lactone **8**, apart from the presence of γ-lactone ring (absorption bands at 1778 and 1199 cm^−1^), a strong band at 3435 cm^−1^ from OH group was also detected. In determining the detailed structure of this compound, it was very helpful to compare its ^1^H NMR data with those of the hydroxylactone obtained from benzaldehyde [27], which is a structural analog containing a methyl group at C-6 instead of a phenyl ring (Table 1). Based on the far-reaching similarities in the chemical shifts of the selected protons and the coupling constants found in the individual multiplets, particularly between H-4 and H-5 (*J* = 6.3 Hz), it was proven that product **8** is also a *trans* isomer of δ-hydroxy-γ-lactone.

**Scheme 3:**
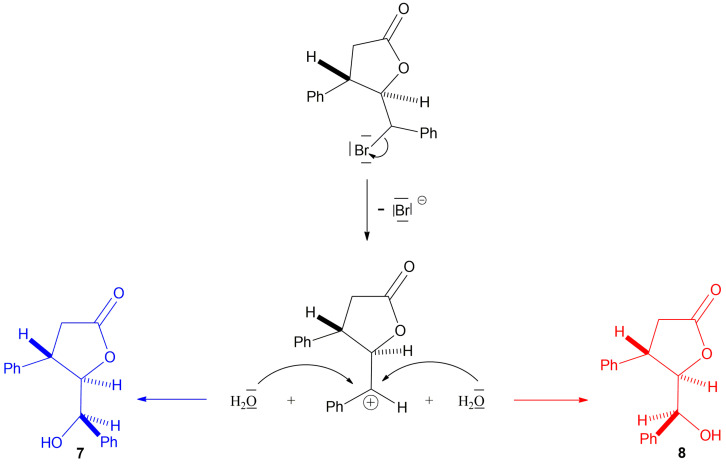
Proposed mechanism of formation of two diastereoisomeric *trans* δ-hydroxy-γ-lactones **7** and **8** from δ-bromo-γ-lactone.

**Figure 4:**
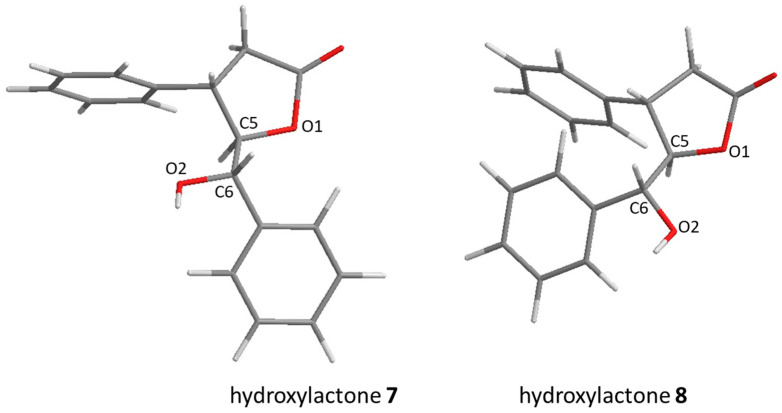
Drawings of diastereoisomeric hydroxylactones **7** and **8** showing the locations of OH group and C-O bond of lactone ring in the relation to C5-C6 bond.

In both isolated hydroxylactones **7** and **8**, a significant difference was observed for chemical shift of proton H-6; the doublet from this proton was located at 4.76 ppm on the spectrum of compound **8** but shifted downfield to 5.13 ppm on the spectrum of lactone **7**. This can be explained by the shorter distance between proton H-6 and the alkoxy oxygen of lactone ring (Figure 3) which exerts more deshielding effect on H-6 in the molecule of lactone **7**. For lactone **8**, this effect is weaker which must be caused by a longer distance between H-6 and O1. The obtained data showed that both *trans* δ-hydroxy-γ-lactones **7** and **8** are diastereoisomers on C-6, differing in the position of the OH group in the relation to the C-O bond of the lactone ring. Formation of two products during bromolactonization of ester **3** can be explained by low stability of C-Br bond in a benzylic position of initially formed *trans* δ-bromo-γ-lactone and nucleophilic substitution occurring according to the S_N_1 mechanism (Scheme 3). In the reaction conditions after dissociation of bromine, the formed carbocation reacts easily with water to form two diastereoisomeric hydroxylactones **7** and **8**. As the water molecule can approach both sides of planar carbocation, in lactone **7**, the OH group and C-O bond of the lactone ring reside on opposite faces of the plane defined by the C5-C6 bond (location *anti* in which torsion angle O1-C5-C6-O2 is in the range of 150–180°), whereas in the lactone **8**, they are situated on the same faces of this plane (location *syn* in which torsion angle O1-C5-C6-O2 is in the range of 0–30°) (Figure 4). These structural features cause the difference in chemical shift of H-6 observed on ^1^H NMR spectra as described above.

### 2.2. Biotransformations of Bromolactone ***5***

The biotransformation of halolactones has been investigated in our research group for many years since it is an alternative to produce new lactone derivatives, usually difficult to obtain by chemical synthesis. γ-Bromo-δ-lactone **5** was also subjected to biotransformation with whole cells of filamentous fungi and yeasts. The preliminary screening showed that 12 strains transformed the substrate to only one product and let us estimate the effectiveness of used strains by comparing the substrate conversions and times of transformation required to achieve the highest conversion (Table 2).

Among tested strains, the lowest conversion (56%) was observed for *A. niger* MB after 14 days of process (entry 1). *A. niger* 13/33 converted the substrate in 74% after 14 days (entry 2) whereas the conversion observed in *A. niger* 13/5 and *A. niger* KB cultures was 88 and 85% after 3 or 14 days, respectively (entries 3, 4). Complete transformation of lactone **5** into the product was achieved for eight strains: in the case of *A. niger* SBJ and *A. niger* SBP after 14 days (entries 5, 6), in the case of yeasts *R. marina* AM77 and fungal strains *P. chrysogenum* AM 112, *P. chermesinum* AM 113, *A. glauca* AM 254 after 10 days (entries 7–10). The most effective biocatalysts were *P. frequentans* AM 359, and *A. niger* CH 11/21 which transformed substrate completely after 7 days of incubation (entries 11, 12).

**Scheme 4:**
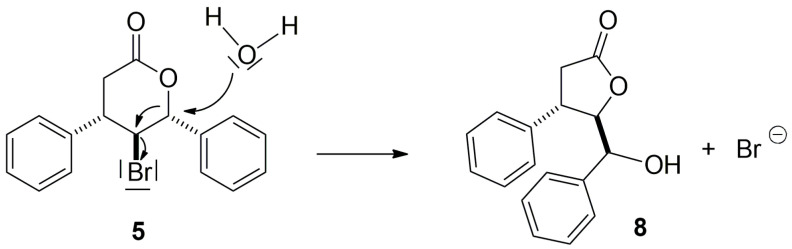
Proposed mechanism of formation of δ-hydroxy-γ-lactone **8** from γ-bromo-δ-lactone **5** in the culture of *Penicillum frequentans* AM 359.

To isolate and establish the structure of a product, biotransformation of δ-bromo-γ-lactone **5** (100 mg) was carried out using *P. frequentans* AM 359. Spectral data of this product, obtained in 88% isolated yield, were fully consistent with those obtained for δ-hydroxy-γ-lactone **8** formed during the bromolactonization of ester **3**. The postulated mechanism of the formation of lactone **8** catalyzed by fungi suggests a tandem dehalogenation-translactonization process involving two simultaneous nucleophilic substitutions: bromine atom at C-5 is substituted by a carboxylic anion released as a result of nucleophilic attack of water at C-6. According to the mechanism of nucleophilic substitution S_N_2, approaching the water molecule from the side opposite to the broken C-O bond in the lactone ring results in the formation of only one isomer of *trans* δ-hydroxy-γ-lactone (**8**) (Scheme 4).

A different course of biotransformation of δ-bromo-γ-lactone **5** was observed in the cultures of *A. cylindrospora* AM 336 and *D. ignaria* KCH 6670 (Table 3).

**Scheme 5:**
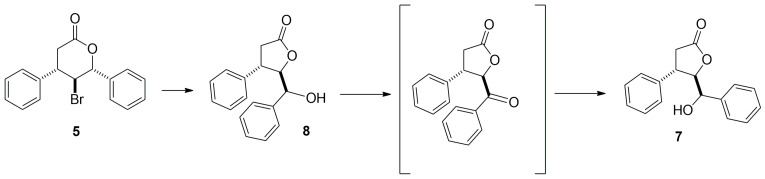
Transformation of γ-bromo-δ-lactone **5** to δ-hydroxy-γ-lactone **8** and subsequent formation of δ-hydroxy-γ-lactone **7** via reversible oxidation/reduction in the culture of *A. cylindrospora* AM 336 and *D. igniaria* KCH 6670.

Analysis of the composition of the products mixtures by GC showed the presence of two isomeric hydroxylactones **7** and **8**. The percentage composition of the reaction mixtures indicated that initially only hydroxylactone **8** was formed and only after 3 or 7 days of the process the formation of hydroxylactone **7** was observed, the amount of which gradually increased at the expense of lactone **8**. Such a course of biotransformation can be explained by the fact that the studied strains possess a dehydrogenase catalyzing a reversible oxidation of hydroxylactone **8** to the corresponding δ-keto-γ-lactone and fast reduction of the carbonyl group leading to the formation of diastereoisomeric hydroxylactone **7** (Scheme 5). A similar reversible oxidation/reduction activity has been described for the fungi-mediated biotransformations of alkylsubstituted cyclohexanones [28] or chalcones [29].

The most frequent transformations of halolactones observed in our earlier studies were hydroxylation of C-H bond in an unactivated position and/or hydrolytic dehalogenation. Piperitone-derived bicyclic δ-halo-γ-lactones were hydroxylated, among others, by *A. cylindrospora* 336 and *A. glauca* AM 254 in none-activated methine carbon at the isopropyl substituent [30]. Hydroxylation of an isopropyl group was also observed during biotransformation of 3-methylcrotonaldehyde-derived δ-iodo-γ-lactone and γ-iodo-δ-lactone using *Botrytis cinerea* AM 235 to afford hydroxyderivatives with tertiary hydroxy group [31]. δ-Iodo- and δ-bromo-γ-lactones derived from β-cyclocitral were hydroxylated in an inactivated position C-5 of cyclohexane ring by *A. cylindrospora* AM 336, their analog with chlorine atom was also transformed to 3-hydroxyderivative [20].

Hydrolytic dehalogenation of halolactones catalyzed by *A. cylindrospora* AM 336 according to the S_N_2 mechanism was observed for bicyclic δ-iodo-γ-lactones with unsubstituted, 4-methyl-, 4,4-dimethyl-, and 5,5-dimethylsubstituted cyclohexane ring as well as for β-phenyl-δ-iodo-γ-lactone. The process was highly stereospecific and the hydroxy group was introduced into the molecule from the opposite site to the leaving halogen atom which was clearly proven by ^1^H NMR and X-ray data [32].

The process of dehalogenation via S_N_2 mechanism with simultaneous translactonization leading to the formation of δ-hydroxy-γ-lactone, analogous to that observed in this work, was also reported during incubation of 3-methylcrotonaldehyde-derived γ-bromo-δ-lactone and γ-chloro-δ-lactone with *Fusarium culmorum* AM 3/1 and *Rhodotorula rubra* AM4, respectively [33]. Another case of tandem translactonization-dehalogenation reaction sequence was observed during biotransformation of δ-iodo-γ-lactone with 4,4-dimethylsubstituted cyclohexane ring by *A. cylindrospora* AM 336. In this case, the hydroxy group introduced by the fungi in the first step of transformation was involved in the intramolecular translactonization followed by immediate nucleophilic substitution by the iodine atom which resulted in the formation of γ,δ-epoxy-γ-lactone [32].

### 2.3. Antimicrobial Activity of Chalcone ***1*** and Lactones ***5**–**8***

**Figure 5:**
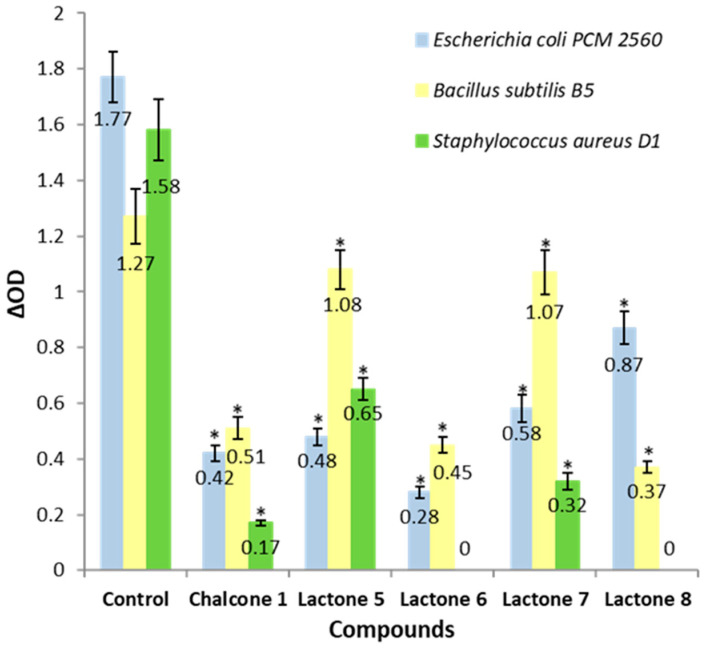
Effect of chalcone **1** and chalcone-derived lactones **5**–**8** on the changes in optical density (ΔOD) for selected bacterial strains. (Control-cultures cultivated in the medium supplemented with DMSO without tested compounds. Results presented as averages for triplicates ± standard deviation. The averages denoted with asterisk are significantly different from the control).

**Figure 6:**
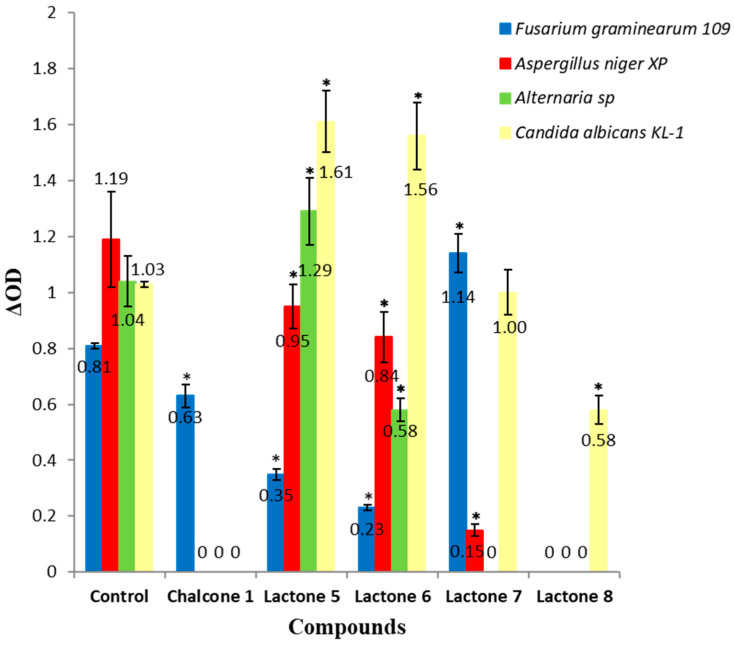
Effect of chalcone **1** and chalcone-derived lactones **5**–**8** on the changes in optical density (ΔOD) for selected filamentous fungi and yeast (Control-cultures cultivated in the medium supplemented with DMSO without tested compounds. Results presented as averages for triplicates ± standard deviation. The averages denoted with asterisk are significantly different from the control).

Antibacterial and antifungal activity of chalcone **1** and lactones **5**–**8** were assessed based on duration of lag-phase (Table 4 and Table 5) and changes in the optical density (ΔOD) of microorganisms growing in the presence of tested compounds (Figure 5 and Figure 6). The ability of a compound to limit the growth of a microorganism is manifested by a prolonged lag phase and/or a significantly lower biomass growth, expressed as ΔOD. Values of these two parameters were compared with those measured for microbial cultures growing without the studied compounds (control cultures). With complete inhibition of the microorganism’s growth, there is no logarithmic growth phase, nor can the duration of the lag phase be determined; these cases are marked in Table 4 and Table 5 as “not determinable″. The tests were carried out for three strains of pathogenic bacteria (*Escherichia coli, Bacillus subtilis, Staphylococcus aureus*), three strains of filamentous fungi (*Fusarium graminearum, Aspergillus niger, Alternaria* sp.), and one strain of yeast (*Candida albicans*). Activity of the compounds was tested at the concentration of 0.1% in dimethyl sulfoxide (10 μL, *w*/*v*).

The results presented in Table 4 and Figure 5 indicate that for all bacterial strains the inhibitory effect of the studied compounds was observed. The highest activity was observed for bromolactone **6** and hydroxylactone **8** against *S. aureus* whose growth was completely inhibited. Relatively high activity towards this strain was also observed for bromolactone **5** and chalcone **1**. In the case of *B. subtilis*, noticeable inhibitory properties were found for chalcone **1**, bromolactone **6**, and hydroxylactone **8** and the least active were bromolactone **5** and hydroxylactone **7**. The most resistant bacterial strain was *E. coli* since in this case, only chalcone **1** significantly extended the lag-phase and mostly decreased biomass growth. Tested lactones influenced only on the latter parameter, and the lowest ΔOD was detected for bromolactone **6**.

The compound with highest antifungal activity (Table 5, Figure 6) turned out to be hydroxylactone **8** which completely inhibited the growth of *F. graminearum*, *A. niger*, and *Alternaria* sp. and was highly active towards yeast *C. albicans.* Its isomer hydroxylactone **7** also totally inhibited the growth of *Alternaria* sp. and significantly limited the growth of *A. niger* but no activity of this compound was found against *F. graminearum* and *C. albicans*. Complete inhibition of microbial growth of *A. niger*, *Alternaria* sp., and *C. albicans* as well as significant limitation of the growth of *F. graminearum* was also observed for chalcone **1.** For bromolactone **6,** noticeably high inhibitory activity was found towards *F. graminearum* and *Alternaria* sp. and lower against *A. niger*, whereas diastereoisomeric bromolactone **5** was particularly active against *F. graminearum* and little active against *A. niger*. Interestingly, a few cases growth-promoting properties of tested compounds were found as ΔOD values were higher than those determined in control cultures. This phenomenon was particularly observed for bromolactones **6** and **5** incubated with *C. albicans*. The latter also promoted the growth of *Alternaria* sp. and hydroxylactone **7** slightly stimulated the growth of *F. graminearum*.

Considering the effect of the introduction of lactone ring into the chalcone scaffold on the antimicrobial activity one can see that a markedly higher activity in comparison with chalcone **1** was observed in the tests against *S. aureus* (Table 4, Figure 5) and *F. graminearum* (Table 5, Figure 6) for bromolactone **6** and hydroxylactone **8.** In the tests against the latter strain, a clear increase in the activity was also observed for bromolactone **5**. Compared to chalcone **1,** lower ΔOD values were observed for bromolactone **6** against *E. coli* and hydroxylactone **8** against *B. subtlilis* (Figure 5) although the durations of lag-phase determined in these tests were much shorter than those obtained for chalcone **1** (Table 4). In the tests against *Alternaria* sp., the activities of hydroxylactones **7** and **8** were as high as the ones found for chalcone **1**; the same situation was observed comparing the activity of chalcone **1** and hydroxylactone **8** against *A. niger.* (Table 5, Figure 6). In other cases, the antifungal activities of lactones were lower than activity of chalcone **1**.

## 3. Materials and Methods

### 3.1. Chemicals

Chalcone (purity 97%), triethyl orthoacetate (97%), sodium borohydride (99%), and *N*-bromosuccinimide (NBS, ≥ 95%) were purchased from Sigma-Aldrich (St. Louis, MO, USA). Other chemicals were of analytical grade (Chempur, Piekary Śląskie, Poland). Silica gel for column chromatography (Kieselgel 60, 230–400 mesh) was purchased from (Merck, Darmstadt, Germany).

### 3.2. Microorganisms Used for Biotransformation of Bromolactone ***5***

Strains of bacteria, yeast, and filamentous fungi used in biotransformations are listed in Table 2 and Table 3. *Aspergillus niger* 13/5 and 13/33 came from the University of Life Sciences in Lublin, *A. niger* CH 11/21, MB, SBJ, and SBP from Wroclaw University of Economics and Bussiness, *A. niger* KB from the Department of Biotechnology and Food Microbiology, Wroclaw University of Environmental and Life Sciences, *D.ignaria* KCH 6670 from Department of Chemistry, Wroclaw University of Environmental and Life Sciences. Strains with abbreviation AM came from Wroclaw Medical University. Microorganisms were stored on Sabouraud agar slants, pH 5.7, containing 1% peptone, 4% glucose, and 8% agar at 4 °C.

### 3.3. Analysis

Analytical Thin Layer Chromatography was performed on silica gel-coated aluminum plates (DC-Alufolien Kieselgel 60 F_254_, Merck, Darmstadt, Germany) using a solution of 1% Ce(SO_4_)_2_ and 2% H_3_[P(Mo_3_O_10_)_4_] × H_2_O in 10% H_2_SO_4_ as a visualizing agent.

The progress of chemical reactions and biotransformations was checked by Gas Chromatography on an Agilent Technologies 6890N instrument with a flame ionization detector (FID) and hydrogen as a carrier gas. Compounds were analyzed on capillary column DB-5HT (30 m × 0.32 mm × 0.10 µm) using temperature program as follows: injector 200 °C, detector 280 °C, column temperature: 140 °C, 140–360 °C (rate 30 °C/min), and 360 °C (hold 1 min).

Nuclear magnetic resonance spectra (^1^H NMR, ^13^C NMR, DEPT 135, ^1^H-^1^H COSY, ^1^H-^13^C HSQC, ^1^H-^13^C HMBC) were performed for samples in CDCl_3_ solutions (99.8% D) on Bruker Avance II 600 MHz spectrometer (Bruker, Rheinstetten, Germany). Signals of residual solvent (δ_H_ = 7.26, δ_C_ = 77.0) were references for chemical shifts. Infrared spectroscopy (IR) spectra were determined using Mattson IR 300 Thermo Nicolet spectrophotometer using KBr pellets. High-resolution mass spectra (HRMS) were recorded on a Bruker micrOTOF-Q II system using electron spray ionization (ESI) technique.

Single-crystal X-ray diffraction data were collected at 293 K (**5**), 100 K (**6**), and 150 K (**7**) on Xcalibur (Sapphire2 CCD detector for **5** and **7** or Onyx CCD detector for **6** κ-geometry diffractometers using *Mo* K*α* (**5** and **7**) or *Cu* K*α* radiation (**6**). Data reduction and analysis were carried out with the CrysAlis Pro programs (CrysAlis PRO. Versions: 1.171.36.28 or 1.171.33.66, currently Rigaku Oxford Diffraction, 2020). The structures were solved by direct methods and refined with the full-matrix least-squares technique using the *SHELXS* [34] and *SHELXL* [35] programs. Non-hydrogen atoms were refined with anisotropic displacement parameters. All H atoms were placed at calculated positions. Before the last cycle of refinement, all H atoms were fixed and were allowed to ride on their parent atoms.

Crystal data for **5**, **6,** and **7** reported in this paper have been deposited with the Cambridge Crystallographic Data Centre (CCDC) as supplementary publication numbers 2,254,514, 2,254,515 and 2,254,516, respectively. Copies of the data can be obtained, free of charge, on application to CCDC, 12 Union Road, Cambridge CB12 1EZ, UK (fax +44-1223-336033 or e-mail: deposit@ccdc.cam.ac.uk).

Uncorrected melting points were determined on Boetius apparatus.

### 3.4. Synthesis of Lactones ***5**–**8***

#### 3.4.1. Reduction of Chalcone (**1**)

A solution of chalcone (**1**) (10.2 g, 49 mmol) in 100 mL of methanol was placed in an ice bath on a magnetic stirrer. Then, an aqueous solution (10 mL) of sodium borohydride (2.35 g, 0.049 mol) was added dropwise and the reaction mixture was stirred by 6 h and after that it was transferred into a separatory funnel, diluted with 25 mL of hot water and the product was extracted with methylene chloride (3 × 40 mL). The combined organic layers were washed with saturated sodium chloride solution until neutral and dried with anhydrous MgSO_4_. The organic solvent was evaporated on a vacuum evaporator to obtain pure alcohol **2**.

(*E*)-1,3-Diphenylprop-2-en-1-ol (**2**): yield 97% (9.91 g), white crystals; mp 44–45 °C (lit. [22] 49 °C); ^1^H NMR (600 MHz, CDCl_3_) δ: 2.17 (s, 1H, OH), 5.39 (d, 1H, *J* = 6.5 Hz, H-1), 6.39 (dd, *J* = 15.9 and 6.5 Hz, H-2), 6.70 (d, *J* = 15.9 Hz, 1H, H-3), 7.21–7.46 (m, 10H, 2 x -C_6_H_5_); ^13^C NMR (150 MHz, CDCl_3_) δ: 75.21 (C-1), 126.46, 126.72, 127.19 and 128.66 (C-2′, C-3′, C-4′, C-5′, C-6′, C-2″, C-3″,C-4″, C-5″, and C-6″), 130.61 (C-3), 131.67 (C-2), 136.66 (C-1′), 142.92 (C-1″); IR (KBr, cm^−1^): 3350 (s), 3026 (m), 1492 (s), 1448 (s), 1319 (m), 1012 (s), 965 (s), 744 (s), 694 (s)

#### 3.4.2. Claisen Rearrangement of Alcohol **2**

A mixture of alcohol **2** (8.63 g; 41 mmol), triethyl orthoacetate (60 mL; 0.33 mol), and propionic acid (two drops) was heated at 138 °C in a two-necked round-bottom flask fitted with a distillation cap. When the reaction was finished (13 h, TLC, GC), the excess of triethyl orthoacetate was distilled off and the crude product was purified by column chromatography (hexane:acetone, 40:1) to afford known [36] ester **3**.

(*E*)-Ethyl 3,5-diphenylpent-4-enoate (**3**): yield 70% (8.05 g), white crystals, mp 30–31 °C; ^1^H NMR (600 MHz, CDCl_3_) δ: 1.17 (t, *J* = 7.1 Hz, 3H, -OCH_2_CH_3_), 2.79 (dd, *J* = 15.0 and 7.5 Hz, 1H, one of CH_2_-2), 2.86 (dd, *J* = 15.0 and 8.0 Hz, 1H, one of CH_2_-2), 4.04 (m, 1H, H-3), 4.08 (quartet, *J* = 7.1 Hz, 2H, -OCH_2_CH_3_), 6.34 (dd, *J* = 15.9 and 6.9 Hz, 1H, H-4), 6.44 (d, *J* = 15.9 Hz, 1H, H-5), 7.17–7.35 (m, 10H, 2 x -C_6_H_5_); ^13^C NMR (150 MHz, CDCl_3_) δ: 14.33 (-OCH_2_CH_3_), 40.93 (C-2), 45.26 (C-3), 60.59 (-OCH_2_CH_3_), 126.40, 126.89, 127.46, 127.70, 128.61 and 128.78 (C-2′, C-3′, C-4′, C-5′, C-6′, C-2″, C-3″, C-4″, C-5″, C-6″), 130.25 (C-5), 132.11 (C-4), 137.24 (C-1″), 142.74 (C-1′), 171.90 (C-1); IR (KBr, cm^−1^): 1723 (s), 1235 (s), 967 (m), 750 (s), 694 (s).

#### 3.4.3. Hydrolysis of Ester **3**

Ester **3** (1.5 g, 5 mmol) was heated at reflux in 30 mL of 2.5% ethanolic NaOH solution. After standard workup of the reaction mixture [37], known [23] acid **4** was obtained.

(*E*)-3,5-diphenylpent-4-enoic acid (**4**): yield 81% (1.09 g), white solid; mp 114–115 °C; ^1^H NMR (600 MHz, CDCl_3_) δ: 2.87 (dd, J = 15.7 and 7.4 Hz, 1H, one of CH_2_-2), 2.90 (dd, *J* = 15.7 and 7.8 Hz, 1H, one of CH_2_-2), 4.03 (m, 1H, H-3), 6.33 (dd, *J* = 15.9 and 6.9 Hz, 1H, H-4), 6.44 (d, *J* = 15.9 Hz, 1H, H-5), 7.20–7.35 (m, 10H, 2 x -C_6_H_5_); ^13^C NMR (150 MHz, CDCl_3_) δ: 40.23 (C-2), 44.78 (C-3), 126.44, 126.84, 127.55, 127.67, 128.64 and 128.88 (C-2′, C-3′, C-4′, C-5′, C-6′, C-2″, C-3″, C-4″, C-5″, C-6″), 137.12 (C-1″), 142.44 (C-1′), 176.47 (C-1); IR (KBr, cm^−1^): 2760–3170 (s), 1705 (s), 1265 (s), 966 (s), 934 (m), 746 (s), 697 (s).

#### 3.4.4. Bromolactonization of Acid **4**

A solution of acid **4** (0.104 g, 40 mmol), NBS (0.082 g, 0.46 mmol), and a drop of acetic acid in THF (20 mL) was stirred at room temperature. When the substrate was consumed (36 h, TLC, GC), the reaction mixture was extracted with diethyl ether (3 × 40 mL), followed by washing with saturated NaHCO_3_ solution and brine. The organic extracts were dried with anhydrous MgSO_4_ and the solvent was evaporated on a vacuum evaporator. The crude product was purified by column chromatography (hexane:acetone, 10:1) to afford known [23] bromolactone **5**.

5-*t*-Bromo-4(*r*),6(*c*)-diphenyltetrahydropyran-2-one (**5**): yield 37% (0.051 g), white crystals, mp 176–178 °C, ^1^H NMR (600 MHz, CDCl_3_) δ: 2.90 (dd, *J* = 17.8 and 10.1 Hz, 1H, one of CH_2_-3), 3.19 (dd, *J* = 17.8 and 6.7 Hz, 1H, one of CH_2_-3 ), 3.63 (td, *J* = 10.1 and 6.7 Hz, 1H, H-4), 4.30 (t, *J* = 10.1 Hz, 1H, H-5), 5.47 (d, *J* = 10.1 Hz, 1H, H-6), 7.25–7.45 (m, 10H, 2 x -C_6_H_5_); ^13^C NMR (150 MHz, CDCl_3_) δ: 37.96 (C-3), 47.42 (C-4), 53.64 (C-5), 85.33 (C-6), 127.19 and 127.82 (C-2″, C-6″, C-2′, C-6′), 128.16 (C-4′), 128.68 and 129.25 (C-3″, C-5″, C-3′, C-5′), 129.60 (C-4″), 136.68 and 140.77 (C-1′, C-1″), 169.11 (C-2); IR (KBr, cm^−1^): 3029 (m), 1729 (s), 1249 (s), 1014 (m).

Crystal data for **5**: C_17_H_15_BrO_2_, M = 331.20, monoclinic, *Cc*, a = 25.688(4) Å, b = 23.478(4) Å, c = 10.806(3) Å, b = 114.84(3)°, V = 5914(3) Å^3^, Z = 16, Dc = 1.488 Mg m^−3^, T = 293(2) K, R = 0.1067, wR = 0.2332 (4344 reflections with I > 2σ(I)) for 627 variables, CCDC 2254514.

#### 3.4.5. Bromolactonization of Ester **3**

A solution of ester **3** (4 g, 14 mmol) and NBS (3.4 g, 19 mmol) was dissolved in 150 mL of a mixture THF:H_2_O (13:2) and stirred at room temperature. After 24 h, the reaction mixture was worked up as described in Section 3.4.4. The crude product mixture was separated by column chromatography (hexane: ethyl acetate, 7:1) and the following products were isolated:

5-*t*-Bromo-4(*r*),6(*c*)-diphenyltetrahydropyran-2-one (**5**): yield 78% (3.68 g), physical and spectral data are given in Section 3.4.4.

5-*c*-Bromo-4(*r*),6(*t*)-diphenyltetrahydropyran-2-one (**6**): Yield 3.4% (0.16 g), white crystals, mp 108–110 °C, ^1^H NMR (600 MHz, CDCl_3_) δ: 2.94 (dd, *J* = 17.1 and 4.5 Hz, 1H, one of CH_2_-3), 3.29 -3.39 (m, 2H, one of CH_2_-3 and H-4), 4.71 (t, *J* = 2.5 Hz, 1H, H-5), 5.97 (d, *J* = 2.5 Hz, 1H, H-6), 7.07–7.09 (m, 2H, H-2′ and H-6′), 7.29–7.31 (m, 1H, H-4′), 7.31–7. 34 (m, 2H, H-3′, H-5′), 7.35–7.37 (m, 2H, H-2″and H-6″), 7.39–7.42 (m, 1H, H-4″), 7.46–7.48 (m, 2H, H-3″ and H-5″); ^13^C NMR (150 MHz, CDCl3) δ: 32.14 (C-3), 37.07 (C-4), 56.50 (C-5), 85.63 (C-6), 125.47 (C-2″, C-6″), 127.34 (C-2′, C-6″), 128.06 (C-4′), 128.84 (C-3′, C-5′), 129.06 (C-4′), 129.39 (C-3″, C-5″), 138.00 (C-1′), 138.39 (C-1″), 168.87 (C-2); IR (KBr, cm^−1^): 3027 (w), 1730 (s), 1205 (s), 1071 (m). HRMS: calcd for C_17_H_15_BrO_2_ [2M+Na]^+^: 683.0408, found 683.0370.

Crystal data for **6**: C_17_H_15_BrO_2_, M = 331.20, monoclinic, P2_1_/n, a = 12.931(3) Å, b = 8.564(2) Å, c = 26.569(4) Å, b = 95.22(2)°, V = 2930.1(11) Å^3^, Z = 8, Dc = 1.502 Mg m^−3^, T = 100(2) K, R = 0.0601, wR = 0.1505 (2455 reflections with I > 2σ(I)) for 361 variables, CCDC 2254515.

*trans*-5-(Hydroxyphenylmethyl)-4-phenyldihydrofuran-2-one (**7**): Yield 3% (0.12 g), white crystals, mp 54–55 °C, ^1^H NMR (600 MHz, CDCl_3_) δ: 2.48 (s, 1H, OH), 2.59 (dd, *J* = 18.2 and 5.1 Hz, 1H, one of CH_2_-3), 3.06 (dd, *J* = 18.2 and 10.1 Hz, 1H, one of CH_2_-3), 3.62 (dt, J = 10.1 and 5. 1 Hz, 1H, H-4), 4.74 (dd, *J* = 5.1 and 3.1 Hz, 1H, H-5), 5.13 (d, *J* = 3.1 Hz, 1H, H-6), 6.86–6.88 (m, 2H, H-2′, H-6′), 7.14–7.21 (m, 3H, H-3′, H-4′, H-5′), 7.28 (m, 1H, H-4″), 7.32–7.34 (m, 2H, H-3″, H-5″), 7.36–7.38 (m, 2H, H-2″, H-6″); ^13^C NMR (150 MHz, CDCl_3_) δ: 37.33 (C-3), 39.80 (C-4), 73.91 (C-6), 89.88 (C-5), 126.30 (C-2″, C-6″), 126.67 (C-2′, C-6′), 127.23 (C-4′), 128.33 (C-4″), 128.72 (C-3″, C-5″), 129.06 (C-3′, C-5′), 138.11 (C-1″), 142.23 (C-1′), 176.94 (C-2); IR (KBr, cm^−1^): 3357 (s), 1768 (s), 1245 (s), 1022 (s). HRMS: calcd for C_17_H_16_O_3_ [2M+Na]^+^: 559.2096, found 559.2087.

Crystal data for **7**: C_17_H_16_O_3_, M = 268.30, monoclinic, P2_1_, a = 5.733(2) Å, b = 8.368(2) Å, c = 14.487(3) Å, b = 97.22(2)°, V = 689.5(3) Å^3^, Z = 2, Dc = 1.292 Mg m^−3^, T = 150(2) K, R = 0.0699, wR = 0.1667 (1797 reflections with I > 2σ(I)) for 181 variables, CCDC 2254516.

*trans*-5-(Hydroxyphenylmethyl)-4-phenyldihydrofuran-2-one (**8**): Yield 6% (0.23 g), white crystals, mp 65–67 °C, ^1^H NMR (600 MHz, CDCl_3_) δ: 2.64 (dd, *J* = 18.1 and 7.8 Hz, 1H, one of CH_2_-3), 2.91 (dd, *J* = 18.1 and 9.5 Hz, 1H, one of CH_2_-3), 3.67 (ddd, *J* = 9.5, 7.8 and 6.3 Hz, 1H, H-4), 4.69 (dd, *J* = 6.3 and 4.1 Hz, 1H, H-5), 4.76 (d, *J* = 4.1 Hz, 1H, H-6), 7.07–7.09 (m, 2H, H-2′, H-6′), 7.23–7.31 (m, 6H, H-3′, H-5′, H-4′, H-4″, H-3″, H-5″), 7.32–7.34 (m, 2H, H-2″, H-6″); ^13^C NMR (150 MHz, CDCl_3_) δ: 37.24 (C-3), 42.76 (C-4), 74.65 (C-6), 89.31 (C-5), 127.05 (C-2″, C-6″), 127.08 (C-2′, C-6′), 127.62 (C-4′), 128.71 (C-4″), 129.01 (C-3″, C-5″), 129.22 (C-3′, C-5′), 139.05 (C-1″), 140.52 (C-1′), 175.83 (C-2); IR (KBr, cm^−1^): 3435 (m), 1778 (s), 1199 (m), 1020 (m). HRMS: calcd for C_17_H_16_O_3_ [M+Na]^+^: 291.0997, found 291.0987.

### 3.5. Biotransformations of Bromolactone ***5***

#### 3.5.1. Screening Procedure

The strains were cultivated on rotary shakers (144 rpm) at 25 °C in 300 mL Erlenmayer flasks containing 50 mL of medium (3% glucose, 1% peptone, pH 6.2). After 5 days, 10 mg of bromolactone **5** dissolved in 1 mL of acetone was added to each flask and the incubation of shaken cultures with substrate was continued for 14 days. Biotransformation products were extracted with ethyl acetate after 1, 3, 7, 10, and 14 days. The extracts were dried with anhydrous magnesium sulphate, concentrated on a rotary evaporator, dissolved in methanol, filtered through a syringe filter (13 mm × 0.45 µm), and analyzed by TLC and GC HPLC. The stability of the substrate was also checked under biotransformation conditions, and the pH of the substrates was tested during the processes. In order to identify the metabolites secreted by individual microbial strains, microorganisms were also cultivated without substrate addition. The results of screening procedure are presented in Table 2 and Table 3.

#### 3.5.2. Biotransformation of Bromolactone **5** by *P. frequentans* AM 359

Bromolactone **5** (100 mg dissolved in 10 mL of acetone) was added to the 5-day cultures of *P. frequentans* AM 359 prepared as described in the screening procedure. The culture was shaken in 2L flask with 400 mL of medium. The progress of biotransformation was monitored by GC. After 7 days, the product was extracted with ethyl acetate. The organic fractions were dried with anhydrous MgSO_4_ and the solvent was evaporated on a vacuum evaporator. Column chromatography (hexane:ethyl acetate; 7:1) afforded 71 mg (yield 88%) of pure hydroxylactone **8** with physical and spectral data consistent with those given in Section 3.4.5.

### 3.6. Antimicrobial Activity Assay

Antimicrobial tests were carried out using the strains from the collection of the Department of Biotechnology and Food Microbiology, Wroclaw University of Environmental and Life Sciences: bacteria *Escherichia coli* PCM 2560, *Staphylococcus aureus* D1, and *Bacillus subtilis* B5, filamentous fungi *Fusarium graminearum* 109, *Alternaria* sp., and *Aspergillus niger* XP, and yeast *Candida albicans* KL-1.

The tests were carried out on the automated Bioscreen C system (Automated Growth Curve Analysis System, Lab Systems, Finland) according to the procedure described in our previous paper [21]. Tested compounds were applied as 0.1% solutions in 10 μL of dimethyl sulfoxide (DMSO) (*w/v*). The results presented in Figure 5 and Figure 6 were analyzed using spreadsheet software (Excel 97). Statistics on a completely randomized design were determined using the one-way analysis of variance (ANOVA) procedure at a level of significance set at *p* < 0.050. Dunnett’s test was used to compare the average ΔOD of microorganism growth in the presence of tested compounds relative to the control.

## 4. Conclusions

In this work, synthetic and biocatalytic approach to chalcone-derived lactones was presented. Bromolactonization of ester **3** with NBS carried out in the mixture of THF and water, in addition to the known γ-bromo-δ-lactone **5,** also gives access to three unexpected products (Scheme 2). The first one is diastereoisomeric γ-bromo-δ-lactone **6** in which a bromine at C-5 and a phenyl ring at C-6 occupy the pseudoaxial positions at the six-membered lactone ring. Diastereoisomeric *trans* δ-hydroxy-γ-lactones **7** and **8** differing in the relative configuration of hydroxy group at C-6 were also isolated. Formation of these two hydroxylactones during bromolactonization of ester **3** is the result of S_N_1 type of nucleophilic substitution involving a facilitated dissociation of the bromine at the benzyl position of primarily formed *trans* δ-bromo-γ-lactone and fast reaction of the benzylic carbocation with the water present in the reaction medium (Scheme 3).

Most of the fungal strains used for the biotransformation of γ-bromo-δ-lactone **5** have the ability to transform the substrate to only one product, *trans* δ-hydroxy-γ-lactone **8**, in the tandem dehalogenation-translactonization process involving two simultaneous S_N_2 nucleophillic substitutions (Scheme 4). Only *A. cylindrospora* AM 336 and *D. igniaria* KCH 6670 produced both isomers of δ-hydroxy-γ-lactones **7** and **8** by reversible oxidation/reduction activity (Scheme 5).

Selective formation of *trans* δ-hydroxy-γ-lactone **8** by *P. frequentans*-mediated biotransformation in relatively high yield (88%) is of special interest because this compound exhibited the most potent inhibitory activity towards tested microorganisms. It was particularly active against fungal strains *F. graminearum*, *A. niger*, and *Alternaria* sp. The most positive effect of the lactone moiety introduced into the scaffold of chalcone **1** on the antibacterial activity was demonstrated towards *S. aureus* by δ-hydroxy-γ-lactone **8** and γ-bromo-δ-lactone **6**; a similar effect on the antifungal activity for these two lactones and γ-bromo-δ-lactone **5** was shown towards *F. graminearum*.

## Figures and Tables

**Table 1:** Comparison of selected signals on ^1^H NMR spectra (chemical shifts, coupling constants) for *trans* δ-hydroxy-γ-lactone **8** and its analog derived from benzaldehyde [27].

*trans* δ-hydroxy-γ-lactone derived from benzaldehyde 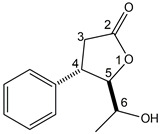	*trans* δ-hydroxy-γ-lactone 8 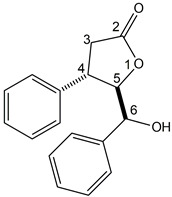	Proton
2.65 (dd, *J* = 18.0, 7.5 Hz) 3.10 (dd, *J* = 18.0, 9.6 Hz)	2.64 (dd, *J* = 18.1, 7.8 Hz) 2.91 (dd, *J* = 18.1, 9.5 Hz)	CH_2_-3
3.75 (ddd, *J* = 9.6, 7.5, 6.3 Hz)	3.67 (ddd, *J* = 9.5, 7.8, 6.3 Hz)	H-4
4.49 (dd, *J* = 6.3, 3.0 Hz)	4.69 (dd, *J* = 6.3, 4.1 Hz)	H-5
4.17 (qd, *J* = 6.3, 3.0 Hz)	4.76 (d, *J* = 4.1 Hz)	H-6

**Table 2:** Compositions of mixtures after biotransformation of bromolactone **5** to one product at the highest substrate conversions.

Entry	Strain	Time (Days)	Substrate 5 (%)	Product 8 (%)
1	*Aspergillus niger* MB	14	44	56
2	*Aspergillus niger* 13/33	14	26	74
3	*Aspergillus niger* 13/5	3	12	88
4	*Aspergillus niger* KB	14	15	85
5	*Aspergillus niger* SBJ	14	0	100
6	*Aspergillus niger* SBP	14	0	100
7	*Rhodotorula marina* AM 77	10	0	100
8	*Penicillum chrysogenum* AM 112	10	0	100
9	*Penicillum chermesinum* AM 113	10	0	100
10	*Absidia glauca* AM 254	10	0	100
11	*Penicillum frequentans* AM 359	7	0	100
12	*Aspergillus niger CH* 11/21	7	0	100

**Table 3:** The course of biotransformation of bromolactone **5** by *A. cylindrospora* AM 336 and *D. igniaria* KCH 6670.

Strain	Time (Days)	Substrate 5 (%)	Product 8 (%)	Product 7 (%)
*Absidia cylindrospora* AM 336	1	47	53	0
	3	6	89	5
	7	3	74	23
	10	2	68	30
	14	2	54	44
*Didymosphearia igniaria* KCH 6670	1	24	76	0
	3	5	95	0
	7	0	95	5
	10	0	92	8
	14	0	83	17

**Table 4:** Effect of chalcone **1** and chalcone-derived lactones **5**–**8** on the duration of lag-phase of selected bacterial strains.

Compound	*Escherichia coli* PCM 2560	*Bacillus subtilis* B5	*Staphylococcus aureus* D1
**1**	21.5 h	27 h	12.5 h
**5**	4 h	8.5 h	19.5 h
**6**	2.5 h	27.5 h	n.d. ^1^
**7**	3 h	6 h	5 h
**8**	3 h	8.5 h	n.d.
Control ^2^	2 h	3 h	4 h

^1^ n.d.—not determinable; ^2^ control—cultures cultivated in the medium supplemented with DMSO without tested compounds.

**Table 5:** Effect of chalcone **1** and chalcone-derived lactones **5**–**8** on the duration of lag-phase of selected filamentous fungi and yeast.

Compound	*Fusarium* *graminearum* 109	*Aspergillus* *niger* XP	*Alternaria* sp.	*Candida* *albicans* KL-1
**1**	21.5 h	n.d. ^1^	n.d.	n.d.
**5**	32 h	14.5 h	20 h	11 h
**6**	55.5 h	22 h	46.5 h	5.5 h
**7**	13 h	37 h	n.d.	6.5 h
**8**	n.d.	n.d.	n.d.	28 h
Control ^2^	12.5 h	12.5 h	9 h	8 h

^1^ n.d.—not determinable; ^2^ control—cultures cultivated in the medium supplemented with DMSO without tested compounds.

## Data Availability

The data presented in this study are available on request from the corresponding author.

## Sample Availability

Samples of the compounds are available from the authors.

## References

[1] Zhuang C., Zhang W., Sheng C., Zhang W., Xing C., Miao Z. (2017). Chalcone: A Privileged structure in medicinal chemistry. Chem. Rev..

[2] Salehi B., Quispe C., Chamkhi I., El Omari N., Balahbib A., Sharifi-Rad J., Bouyahya A., Akram M., Iqbal M., Docea A.O. (2021). Pharmacological properties of chalcones: A review of preclinical including molecular mechanisms and clinical evidence. Front. Pharmacol..

[3] Sahu K.N., Balbhadra S.S., Choudhary J., Kohli D.V. (2012). Exploring Pharmacological Significance of Chalcone Scaffold: A Review. Curr. Med. Chem..

[4] Singh P., Anand A., Kumar V. (2014). Recent developments in biological activities of chalcones: A mini review. Eur. J. Med. Chem..

[5] Elkanzi N.A.A., Hrichi H., Alolayan R.A., Derafa W., Zahou F.M., Bakr R.B. (2022). Synthesis of chalcones derivatives and their biological activities: A review. ACS Omega.

[6] Constantinescu T., Lungu C.N. (2021). Anticancer activity of natural and synthetic chalcones. Int. J. Mol. Sci..

[7] Xu M., Wu P., Shen F., Ji J., Rakesh K.P. (2019). Chalcone derivatives and their antibacterial activities: Current development. Bioorg. Chem..

[8] Lahtchev K.L., Batovska D.I., Parushev S.P., Ubiyvovk V.M., Sibirny A.A. (2008). Antifungal activity of chalcones: A mechanistic study using various yeast strains. Eur. J. Med. Chem..

[9] Okolo E.N., Ugwu D.I., Ezema B.E., Ndefo J.C., Eze F.U., Ezema C.G., Ezugwu J.A., Ujam O.T. (2021). New chalcone derivatives as potential antimicrobial and antioxidant agent. Sci. Rep..

[10] Vásquez-Martínez Y.A., Osorio M.E., San Martín D.A., Carvajal M.A., Vergara A.P., Sanchez E., Raimondi M., Zacchino S.A., Mascayano C., Torrent C. (2019). Antimicrobial, anti-inflammatory and antioxidant activities of polyoxygenated chalcones. J. Braz. Chem. Soc..

[11] Kozłowska J., Potaniec B., Zarowska B., Anioł M. (2018). Microbial transformations of 4′-methylchalcones as an efficient method of obtaining novel alcohol and dihydrochalcone derivatives with antimicrobial activity. RSC Adv..

[12] Kuttithodi A.M., Nikhitha D., Jacob J., Narayanankutty A., Mathews M., Olatunji O.J., Rajagopal R., Alfarhan A., Barcelo D. (2022). Antioxidant, antimicrobial, cytotoxicity, and larvicidal activities of selected synthetic bis-chalcones. Molecules.

[13] Gupta D., Jain D.K. (2015). Chalcone derivatives as potential antifungal agents: Synthesis, and antifungal activity. J. Adv. Pharm. Technol. Res..

[14] Liaras K., Geronikaki A., Glamočlija J., Ćirić A., Soković M. (2011). Thiazole-based chalcones as potent antimicrobial agents. Synthesis and biological evaluation. Bioorg. Med. Chem..

[15] Siddiqui Z.N., Praveen S., Musthafa T.N.M., Ahmad A., Khan A.U. (2012). Thermal solvent-free synthesis of chromonyl chalcones, pyrazolines and their in vitro antibacterial, antifungal activities. J. Enzym. Inhib. Med. Chem..

[16] López S.N., Castelli M.V., Zacchino S.A., Domínguez J.N., Lobo G., Charris-Charris J., Cortés J.C.G., Ribas J.C., Devia C., Rodríguez A.M. (2001). In vitro antifungal evaluation and structure-activity relationships of a new series of chalcone derivatives and synthetic analogues, with inhibitory properties against polymers of the fungal cell wall. Bioorg. Med. Chem..

[17] Skrobiszewski A., Gładkowski W., Walczak P., Gliszczyńska A., Maciejewska G., Klejdysz T., Nawrot J., Wawrzeńczyk C. (2015). Synthesis of β-aryl-γ-lactones and relationship: Structure–Antifeedant and antifungal activity. J. Chem. Sci..

[18] Mazur M., Skrobiszewski A., Gladkowski W., Podkowik M., Bania J., Nawrot J., Klejdysz T., Wawrzeńczyk C. (2016). Lactones 46. Synthesis, antifeedant and antibacterial activity of γ-lactones with a p-methoxyphenyl substituent. Pest Manag. Sci..

[19] Włoch A., Stygar D., Bahri F., Bażanów B., Kuropka P., Chełmecka E., Pruchnik H., Gładkowski W. (2020). Antiproliferative, antimicrobial and antiviral activity of β-aryl-δ-iodo-γ-lactones, their effect on cellular oxidative stress markers and biological membranes. Biomolecules.

[20] Mazur M., Gładkowski W., Podkowik M., Bania J., Nawrot J., Białońska A., Wawrzeńczyk C. (2014). Lactones 43. New biologically active lactones: β-cyclocitral derivatives. Pest Manag. Sci..

[21] Gładkowski W., Siepka M., Janeczko T., Kostrzewa-Susłow E., Mazur M., Żarowska B., Łaba W., Maciejewska G., Wawrzeńczyk C. (2019). Synthesis and antimicrobial activity of methoxy-substituted γ-oxa-eε-lactones derived from flavanones. Molecules.

[22] Silva V.D., Stambuk B.U., da Nascimento M.G. (2010). Efficient chemoselective biohydrogenation of 1,3-diaryl-2-propen-1-ones catalyzed by *Saccharomyces cerevisiae* yeasts in biphasic system. J. Mol. Catal. B Enzym..

[23] Liu D., Yu X., Huang L. (2013). Novel concise synthesis of (-)-clausenamide. Chin. J. Chem..

[24] Obara R., Szumny A., Żołnierczyk A., Olejniczak T., Białońska A., Ciunik Z., Wawrzeńczyk C. (2005). Lactones 17. Synthesis of bicyclic lactones with the methyl- or gem-dimethylcyclopropane system. Pol. J. Chem..

[25] Gładkowski W., Skrobiszewski A., Mazur M., Siepka M., Pawlak A., Obmińska-Mrukowicz B., Białońska A., Poradowski D., Drynda A., Urbaniak M. (2013). Synthesis and anticancer activity of novel halolactones with β-aryl substituents from simple aromatic aldehydes. Tetrahedron.

[26] Gładkowski W., Włoch A., Pawlak A., Sysak A., Białońska A., Mazur M., Mituła P., Maciejewska G., Obmińska-Mrukowicz B., Kleszczyńska H. (2018). Preparation of enantiomeric β-(2’,5’-dimethylphenyl) bromolactones, their antiproliferative activity and effect on biological membranes. Molecules.

[27] Skrobiszewski A., Gładkowski W., Lis M., Gliszczyńska A., Maciejewska G., Klejdysz T., Obmińska-Mrukowicz B., Nawrot J., Wawrzeńczyk C. (2014). Laktony. Cz. XLV. Synteza hydroksylaktonów z pierścieniem aromatycznym oraz ocena ich aktywności antyfidantnej i antyproliferacyjnej. Przem. Chem..

[28] Ratuś B., Gładkowski W., Wawrzeńczyk C. (2009). Lactones 32 [1]. New aspects of the application of *Fusarium* strains to production of alkylsubstituted ε-lactones. Enzyme Microb. Technol..

[29] Janeczko T., Gładkowski W., Kostrzewa-Susłow E. (2013). Microbial transformations of chalcones to produce food sweetener derivatives. J. Mol. Catal. B Enzym..

[30] Mazur M., Grudniewska A., Wawrzeńczyk C. (2015). Microbial transformations of halolactones with *p*-menthane system. J. Biosci. Bioeng..

[31] Grotowska A.K., Wawrzeńczyk C. (2002). Lactones 13: Biotransformation of iodolactones. J. Mol. Catal. B Enzym..

[32] Gładkowski W., Mazur M., Białońska A., Wawrzeńczyk C. (2011). Lactones 35 [1]. Metabolism of iodolactones with cyclohexane ring in *Absidia cylindrospora* culture. Enzyme Microb. Technol..

[33] Żołnierczyk A.K., Anioł M., Wawrzeńczyk C. (2013). Mikrobiologiczna dehalogenacja chloro- i bromo-laktonów. Przem. Chem..

[34] Sheldrick G.M. (2008). A short history of SHELX. Acta Crystallogr. Sect. A Found. Crystallogr..

[35] Sheldrick G.M. (2015). Crystal structure refinement with SHELXL. Acta Crystallogr. Sect. C Struct. Chem..

[36] Zhao J., Ye J., Zhang Y.J. (2013). Stereospecific allyl-aryl coupling catalyzed by in situ generated palladium nanoparticles in water under ambient conditions. Adv. Synth. Catal..

[37] Gładkowski W., Skrobiszewski A., Mazur M., Siepka M., Białońska A. (2015). Convenient chemoenzymatic route to optically active β-aryl-δ-iodo-γ-lactones and β-aryl-γ-iodo-δ-lactones with the defined configurations of stereogenic centers. Eur. J. Org. Chem..

